# Supplementary material for: Understanding General Practitioner and Patient Perceptions Regarding Integration of Non-Pharmacological Interventions in Chronic Non-Cancer Pain Management—A Cross-Sectional Mixed-Methods Study in the RELIEF Project
Source: Diseases. 2025 Jan 28;13(2):34. doi: 10.3390/diseases13020034 (PMC11854548; doi:10.3390/diseases13020034)
Supplement: Supplementary file 1 [file diseases-13-00034-s001.zip › diseases-3369711-supplementary.pdf]

Topics covered during data collection
Supplementary S1: Topics covered in the survey

Survey questionnaire - General practitioner

- Socio-demographic characteristics of participants and practices
- Role in pain management
- Treatment approach
- Prescribing of medication (over the counter; analgesics; opioids)
- Treatment options outside general practice
- Familiarity with relevant guidelines
- Case studies
- Potential elements in a case management program
- Training

Survey questionnaire - Patients

- Current pain situation
- Pain history
- Pain treatment
- Use of analgesics and over the counter products
- Use of non-pharmacological interventions
- Use of health services
- Psychological aspects
- Self-efficacy
- Information received
- Thought experiment
- Socio-demographic characteristics of participants

Topics covered during data collection
Supplementary S2: Topics covered in the interviews

Interview - General practitioner

- Socio-demographic characteristics of participants
- Treatment approach
- Assessment and monitoring
- Knowledge transfer/patient education
- Non-pharmacological interventions
- Rational pharmacotherapy
- Familiarity with relevant guidelines
- Prescribing of opioids
- Potential elements in a case management program
- What else is relevant?

Interview - Patient

- Pain history and experiences with treatment approaches
- Pain treatment in general practice
- Knowledge transfer/patient education
- Psychological aspects
- Use of analgesics and over the counter products
- Use of non-pharmacological interventions
- Social support
- Thought experiment
- Socio-demographic characteristics of participants
